# Two Polycyclic Geranylhydroquinone-Derived Metabolites from Roots of *Arnebia hispidissima* (Lehm.) DC.

**DOI:** 10.3390/molecules19055940

**Published:** 2014-05-09

**Authors:** Atallah F. Ahmed, Hassan-Elrady A. Saad, Eman M. Abd El-Karim

**Affiliations:** 1 Department of Pharmacognosy, College of Pharmacy, King Saud University (KSU), Riyadh 11451, Kingdom of Saudi Arabia; 2 Department of Pharmacognosy, Faculty of Pharmacy, Mansoura University (MU), Mansoura 35516, Egypt

**Keywords:** *Arnebia hispidissima*, arnebacene, arnebidin, arnebin-7, phenolic compounds, geranylhydroquinone

## Abstract

A phytochemical investigation of the least polar organic extracts of *Arnebia hispidissima* (Lehm.) DC. roots has led to the isolation of two unique polycyclic geranylhydroquinone-derived metabolites, arnebacene (**1**) and arnebidin (**2**), along with some known phenolic metabolites *viz*., arnebin-7 (**3**) and vanillic acid (**4**). The chemical identification of the new isolated compounds, including their relative stereochemistry, was achieved via spectroscopic analyses, including 2D NMR, and spectral comparison with related compounds. A biosynthetic pathway is proposed for the new compounds on the basis of their structure-relationship with previously isolated metabolites.

## 1. Introduction

*Arnebia hispidissima* (Lehm.) DC. (Arabian primrose), which belongs to the family Boraginaceae, is widely distributed in the northern Africa area, through Egypt, to northern India [1]. To date, the roots of eight *Arnebia* species were studied phytochemically, and several naphthoquinone metabolites possessing various biological activities, have been isolated [2,3,4,5,6,7]. The root of *A. hispidissima* has been used as a food colorant whereas the flowering shoot has been employed in diseases of the tongue and throat as well as fevers and cardiac disorders [4,8]. Moreover, the organic extracts of *A. hispidissima* exhibited antibacterial [3,9] and antitumor activity [10] which were attributable to the presence of naphthoquinones, triterpenoids, and pyrrolizidine alkaloids. Our phytochemical investigation on the root of *Arnebia hispidissima* growing in Sinai Peninsula, has led to the isolation of new furanohexahydroanthracene-based metabolite arnebacene (**1**) and the arnebin-7-related dimer arnebidin (**2**), along with two known phenolic compounds **3** and **4**. The structures of the new metabolites, including their stereochemistry, were elucidated on the basis of extensive spectroscopic analyses (including 1D and 2D NMR) and by comparison of their spectral data with those of related compounds. Compound **2** is reported herein as a novel heptacyclic arnebin-7 dimer with an unprecedented tricyclo[3.3.0.0^1,3^]octane core.

## 2. Results and Discussion

**Figure 1:**
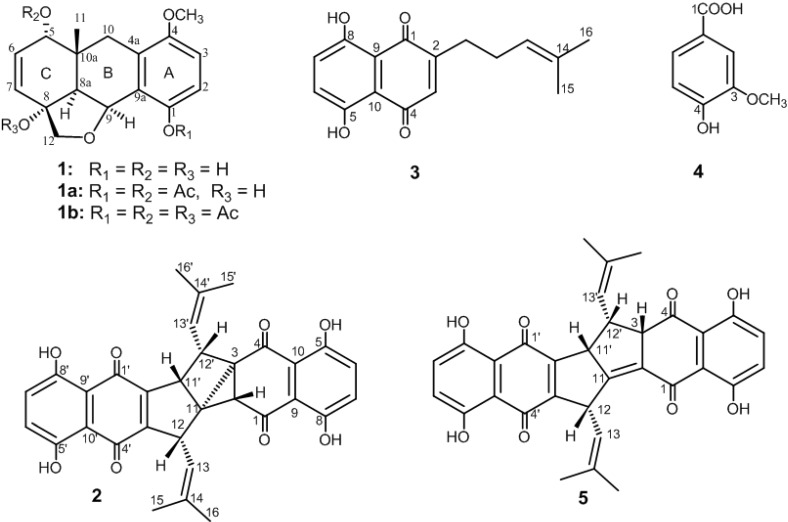
Phenolic constituents (**1**–**4**) isolated from *A. hispidissima* roots and shikometabolin D (**5**).

The dried root of *A. hispidissima* was powdered, sequentially extracted with light petroleum and then MeOH. The MeOH extract was further partitioned with H_2_O/*n*-hexane, H_2_O/CH_2_Cl_2_, and then with H_2_O/EtOAc to yield MHF, MCF, and MEF fractions, respectively. The light petroleum extract and MeOH-derived fractions were separately subjected to chromatographic fractionation and purification, utilizing a series of normal-phased chromatographic systems, to afford compounds **2** and **3** from the light petroleum extract, **1** from MCF, and **4** from MEF (Figure 1).

Compounds **3** and **4** were identified by comparison of their physical and spectroscopic (MS and NMR) data with those of the previously isolated compounds as arnebin-7 [11] (syn. deoxyalkannin [12] or deoxyshikonin [7]) and vanillic acid [13], respectively. Compound **4** is reported herein from genus *Arnebia* for the first time, although it was isolated once from the genus *Onosma* [14] of the same family (Boragenaceae).

**Figure 2:**
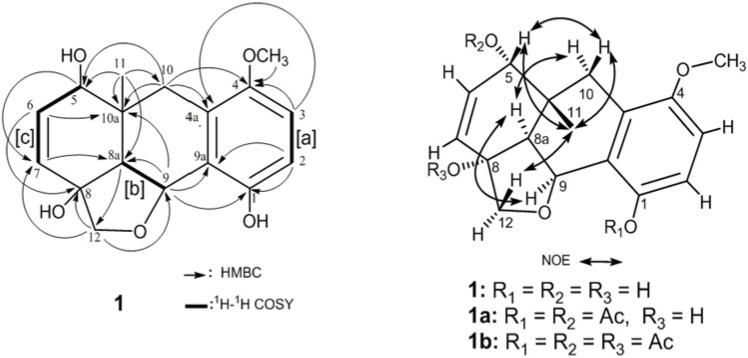
^1^H-^1^H COSY and HMBC correlations in **1** and key NOESY correlations in **1**, **1a** and **2b**.

Compound **1** was isolated as colorless feathery crystals with a molecular formula of C_17_H_20_O_5_, implying eight degrees of unsaturation, as established by the HRFAB-MS *m/z* 304.1307 (M^+^). Its absorption bands in the UV (281 nm) and IR (3356 and 1601 cm^−1^) spectra revealed the presence of a phenolic moiety. The peaks appearing in the in EI-MS at *m/z* 286 [M-H_2_O]^+^ and 239 [M-H_2_O-Me-MeOH]^+^ and in FAB-MS at *m/z* 269 [M-H_2_O+H]^+^ indicated the presence in the structure of **1** of at least two hydroxyl groups, a methyl and a methoxy group. The ^13^C-NMR spectrum displayed 17 carbon signals (Table 1) which were assigned, by the assistance of DEPT spectra, into two methyls, two methylenes, seven methines (including two *sp^3^* oxymethines), and six quaternary carbons (including one *sp^3^* and two *sp^2^* oxygenated carbons). The eight *sp^2^* carbons resonating at δ_C_ 111.4–151.3 indicated the presence of four double bonds. Thus, the remaining four degrees of unsaturation indicated a tetracyclic skeleton for compound **1**. The HMQC spectrum showed δ_H_/δ_C_ correlations at 2.67 (2H, s)/33.2; 3.92 and 4.08 (each 1H, d, *J* = 10.0 Hz)/77.9; 0.85 (3H, s)/19.5; and 3.79 (3H, s)/56.1 attributable to the presence of an isolated methylene (10-CH_2_), an isolated oxymethylene (12-CH_2_), a ring-junction tertiary methyl (10a-CH_3_) and an aromatic methoxy group (4-OCH_3_), respectively. The ^1^H-^1^H COSY experiment established three partial proton spin systems structure (Figure 2) of a 1,2,3,4-tetrasubstituted benzene [a] (δ_H_ 6.77 and 6.80, each 1H, *d*, *J* = 8.8 Hz), a 1,2-disubstituted oxyethylene unit [b] (δ_H_ 2.59 and 5.39, each 1H, *d*, *J* = 6.0 Hz), and a 1-oxyallyl moiety [c] (δ_H_ 6.01, 1H, *d*, *J* = 10.0Hz; 6.11, 1H, *dd*, *J* = 10.0, 6.0 Hz; and 3.84, 1H, *d*, *J* = 6.0 Hz). The linking of the above mentioned three partial structures [a–c], the isolated two methylenes, the tertiary methyl, the methoxy group with the remaining six quaternary carbons in the molecule (Figure 2) was established by the HMBC correlations as follows: the aromatic protons (H-2 and H-3) and the olefinic protons (H-6 and H-7) exhibited *^3^J* correlations to C-9a (qC), C-4a (qC), C-10a (qC) and C-8a (CH), respectively. Since the isolated methylene protons H_2_-10 and the oxymethine proton H-9 were found correlated to C-4a and C-10a and to C-9a and C-8a, respectively, therefore ring B should be fused with rings A and C through C-4a/C-9a and C-10a/C-8a, respectively (Figure 1 and Figure 2). The HMBC correlations observed from upfield shifted protons at δ_H_ 0.85 (3H, *s*) to C-5, C-8a, C-10, and C-10a positioned the single methyl group in the molecule at the ring juncture carbon C-10a. Furthermore, the HMBC correlations exhibited from H_2_-12 to C-8 (δ_C_ 78.8, qC) and C-9 (δ_C_ 73.9, CH) and from H-8a (δ_H_ 2.59, 1H, d, *J* = 6.0 Hz), to C-12 indicated a trisubstituted furan ring to be located at C-8, C-8a, and C-9. Acetylation of compound **1** afforded diacetate and triacetate derivatives **1a** and **1b**. The latter compound **1b** displayed ester carbonyl absorptions at 1770 and 1734 cm^−1^ and no hydroxyl absorptions in the IR spectrum, whereas it exhibited successive ions peaks at *m/z* 370 [M-AcOH]^+^, 310 [M-2AcOH]^+^, and 250 [M-3AcOH]^+^ in the EI-MS due to elimination of three acetoxyl groups. Therefore, the three hydroxyl groups in **1** were determined. The C-1, C-5, and C-8 positions of these hydroxyl groups were suggested by detailed analysis of HMBC correlations of compound **1** (Figure 2). Moreover, the NMR data of **1b** showed an upfield shift at C-1 (Δδ_C_-5.9) and downfield shifts at 5-CH (Δδ_H_ + 1.06 and Δδ_C_ + 0.8) and C-8 (Δδ_C_ + 8.3), relative to those of compound **1**, which further supported the locations of hydroxyl groups to be at C-1, C-5, and C-8, respectively. It is noteworthy to mention that the 2H singlet of the methylene protons at C-10 (δ_H_ 2.67) was transformed into two 1H doublets in the diacetate **1a** (δ_H_ 2.49, H-10α and 2.73, H-10β, each 1H, *d*, *J* = 15.6 Hz) and in the triacetate **1b** (δ_H_ 2.54, H-10α and 2.73, H-10β, each 1H, *d*, *J* = 16.4 Hz) derivatives as a result of acetylation of 5-OH in compound **1**. On the basis of the above findings, the gross structure of compound **1** was thus deduced as illustrated in Figure 2. 

**Table 1:** ^1^H and ^13^C-NMR chemical shifts for **1**.

Atom	1 *^a^*	
	δ_C_	δ_H_
1	149.9 (qC)	
2	113.3 (CH) *^b^*	6.80, *d* (8.8) *^c^*
3	111.4 (CH)	6.77, *d* (8.8)
4	151.3 (qC)	
4a	124.7 (qC)	
5	70.3 (CH)	3.84, *d* (6.0)
6	129.7 (CH)	6.11, *dd* (10.0, 6.0)
7	131.2 (CH)	6.01, *d* (10.0)
8	78.8 (qC)	
8a	49.8 (CH)	2.59, *d* (6.0)
9	73.9 (CH)	5.39, *d* (6.0)
9a	122.0 (qC)	
10	33.2 (CH_2_)	2.67, 2 H, *s*
10a	35.9 (qC)	
11	19.5 (CH_3_)	0.85, 3H, *s*
12	77.9 (CH_2_)	4.08, *d* (10.0), α
		3.92, *d* (10.0), β
4-OMe	56.1 (CH_3_)	3.79, 3H, *s*

^a^ Spectra recorded at 500 MHz in CDCl_3_ for ^1^H and 125 MHz for ^13^C; ^b^ Attached protons were determined by DEPT and HMQC experiments; ^c^ The *J* values are in Hz in parentheses. The chemical shift values (δ_H_/ δ_C_) are in ppm downfield from TMS.

The relative stereochemistry of compound **1** was interpreted from the NOE interactions (Figure 2) observed in the NOESY spectra of **1**, **1a** and **1b**. Supposing the β-orientation of the ring-juncture methyl at C-10a, the significant NOESY correlations observed between Me-11 and H-5 revealed the α-orientation of the 5-OH group in **1**. Although the methine protons H-8a in **1**, **1a**, and **1b** displayed a strong NOE interaction with H-9, both protons lacked NOE cross-peaks with Me-11. The α-orientation for H-8a and H-9 in **1** was thus suggested. One of the methylene protons at C-10 in **1a** (δ_H_ 2.73) or **1b** (δ_H_ 2.73) displayed NOE interactions with Me-11 and H-5 and designated as Hβ-10 whereas the other one (δ_H_ 2.49 in **1a** or δ_H_ 2.54 in **1b**) was found to be NOE correlated with H-8a. The α-orientation for H-8a and consequently for H-9 was thus confirmed. Moreover, one oxymethylene proton at C-12 in **1a** (δ_H_ 3.92) or **1b** (δ_H_ 4.05) exhibited significant NOE response with Me-11 of **1a** or **1b**, respectively, disclosing the β-configuration of the furan ring and thus 8-OH should be α-oriented. From the above findings, the structure and relative configuration of **1** was unambiguously established as (5S*,8R*,8aS*,9S*,10aR*)-1,5,8-trihydroxy-4-methoxy-10a-methyl-8,9-oxymethylene-5,10a,8,8a,9,10-hexahydroanthracene and named arnebacene. 

**Figure 3:**
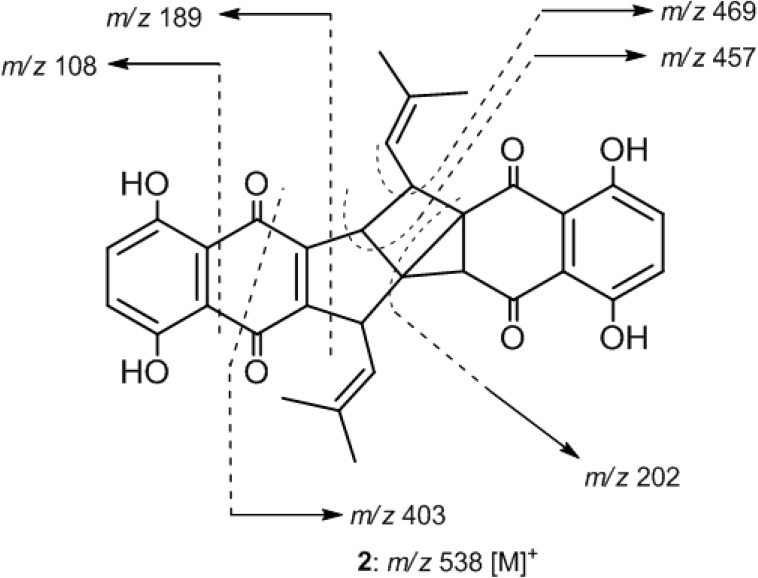
EI-MS fragmentation pattern of **2**.

**Figure 4:**
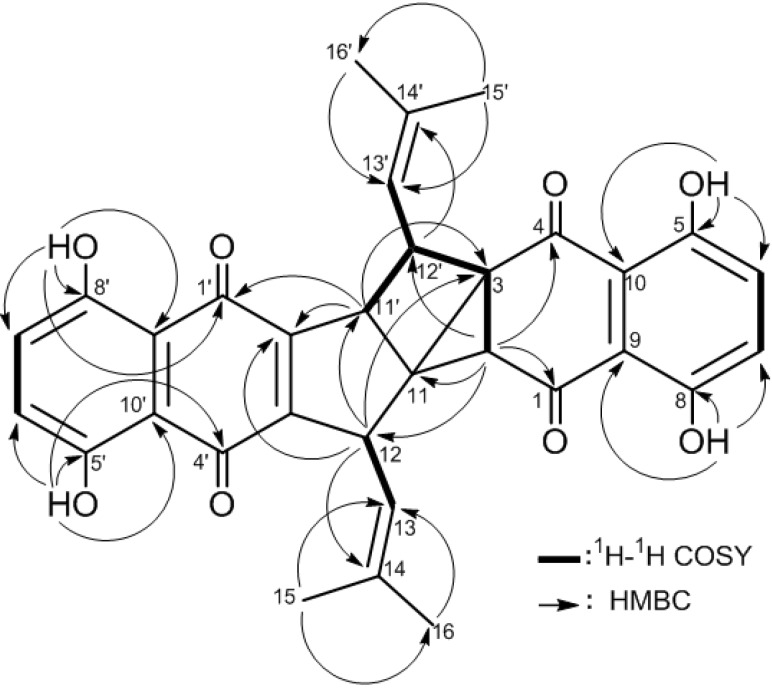
^1^H-^1^H COSY and HMBC correlations for **2**.

Compound **2** was isolated as an orange-red amorphous powder. The molecular ion peak appearing in the EI-MS (*m/z* 538 [M]^+^, 100% abundance) and the NMR spectral data (Table 2) established a molecular formula of C_32_H_26_O_8_. Therefore, twenty degrees of unsaturation were determined for compound **2**. The EI-MS exhibited ion peaks at *m/z* 189 [C_10_H_5_O_4_]^+^, and 108 [C_6_H_4_O_2_]^+^ (Figure 3) diagnostic for naphthoquinone-derived monomers such as arnebin-7 (**3**) [11,15]. The ^1^H-NMR spectrum showed 26 proton signals (Table 2) attributed to four vinylic methyls (δ_H_ 1.54, 1.61, 1.72 and 1.83, 3H each, *d*, *J* = 1.5 Hz), two vinylic methines (δ_H_ 4.39 and 4.63, 1H each, *ddd*, *J* = 8.5, 1.5, 1.5 Hz) and four *sp^3^* methines (δ_H_ 2.68, 1H, *d*, *J* = 2.5 Hz; 3.35, 1H, *ddd*, *J* = 8.5, 4.5, 2.5 Hz; 3.82, 1H, *dd*, J = 8.5, 4.5 Hz and 3.89, 1H, *dd*, *J* = 4.5, 1.5 Hz). The remaining eight proton signals indicated the presence of two 1,2,3,4-tetrasubstituted phenyl moieties (δ_H_ 7.22, 7.25, 7.29 and 7.30, 1H each, *d*, *J* = 8.5 Hz), each possesses 1,4-dihydroxy groups (δ_H_ 12.07, 12.30, 12.40 and 12.50, 1H each, *s*). Moreover, the ^13^C-NMR data (Table 2) displayed 32 carbon signals, indicating the presence of four keto-carbonyls (δ_C_ 182.2–195.5, 4C, each qC) and eighteen *sp^2^* carbons (δ_C_ 111.4–159.1, 12 C, each qC and 116.9–130.0, 6C, each CH) of nine carbon-carbon double bonds, four methyls (δ_C_ 18.6–26.0, 4C, each CH_3_), four *sp^3^* methine (δ_C_ 37.6–45.3, 4C, each CH), and two *sp^3^* quaternary carbons (δ_C_ 49.2 and 38.3, each qC). Comparison of NMR data of **2** with those of arnebin-7 (**3**) (Table 2) suggested compound **2** as a dimer of **3** in which six protons were eliminated. Therefore, compound **2** should possess an heptacyclic structure. Moreover, comparison of ^13^C-NMR data (Table 2) with those of shikometabolin D (**5**), another arnebin-7 dimer produced by anaerobic incubation of shikonin with *Bacteroides fragilis* [15,16], revealed high similarity. The only difference is the disappearance of a tetrasubstituted olefinic carbon-carbon double bond at δ_C_ 117.4 (C-11, qC) and 137.5 (C-2, qC) in **5** and the appearance of two quaternary *sp^3^* carbons at δ 38.3 and 49.2 in **2** instead. Thus, compound **2** differs in the presence of an additional ring, including C-2 and C-11 as ring-juncture carbons. The ^1^H/^1^H correlations observed in the COSY spectrum of **2** (Figure 4) revealed the presence of two consecutive spin-spin system extending from H-12 to H-13 and from H-11' to H-13' through H-12'. On the other hand a long-range ^1^H/^1^H correlation was observed from H-12' (δ_H_ 3.35, 1H, *ddd*, *J* = 8.5, 4.5, 2.5 Hz) to H-2 (δ_H_ 2.68, 1H, *d*, *J* = 2.5 Hz). Thus, the only *sp^3^* quaternary carbons in 2 should be C-11 and C-3. This result was further evidenced from the ^1^H/^13^C long-range correlations observed in the HMBC spectrum (Figure 4 from H-2 to C-11 (δ_C_ 38.3, qC) and from H-11' (δ_H_ 3.89, 1H, *dd*, *J* = 4.5, 1.5 Hz) to C-2 (δ_C_ 45.3, CH) and C-3 (δ 49.2, qC). Therefore, an additional tricyclic ring is formed in the structure of **2**connecting C-2 with both C-3 and C-11 to form a strained bicyclo[2.1.0]pentanyl moiety. Furthermore, other detailed COSY and HMBC correlations (Figure 4) along with EI-MS fragmentation pattern (Figure 3) established the planar structure of compound **2**. 

**Table 2:** ^1^H and ^13^C-NMR chemical shifts for **2** and **3**.

Atom	2 *^a^*		3 *^a^*	
	δ_C_	δ_H_	δ_C_	δ_H_
1	193.1 (qC)			
2	45.3 (CH) *^b^*	2.68, *d* (2.5) *^c^*		
3	49.2 (qC)			
4	195.5 (qC)			
5	156.3 (qC)			
6	129.1 (CH)	7.29, *d* (8.5)		
7	128.9 (CH)	7.30, *d* (8.5)		
8	157.4 (qC)			
9	111.7 (qC)			
10	112.7 (qC)			
11	38.3 (qC)			
12	40.7 (CH)	3.82, *dd* (8.5, 4.5)		
13	116.9 (CH)	4.39, *ddd* (8.5, 1.5, 1.5)		
14	138.6 (qC)			
1'	182.2 (qC)		183.0 (qC)	
2'	144.3 (qC)		151.5 (qC)	
3'	140.0 (qC)		134.5 (CH)	6.84, *s*
4'	182.6 (qC)		183.0 (qC)	
5'	159.1 (qC)		162.3 (qC)	
6'	129.3 (CH)	7.25, *d* (8.5)	131.2 (CH)	7.19, *s*
7'	129.9 (CH)	7.22, *d* (8.5)	130.9 (CH)	7.19, *s*
8'	158.6 (qC)		163.0 (qC)	
9'	112.5 (qC)		111.7 (qC)	
10'	111.4 (qC)		112.0 (qC)	
11'	38.1 (CH)	3.89, *dd* (4.5, 1.5)	26.6 (CH_2_)	2.64, 2H, *ddd* (7.5, 1)
12'	37.6 (CH)	3.35, *ddd* (8.5, 4.5, 2.5)	29.7 (CH_2_)	2.30, 2H, *q*, (13.5, 7.5)
13'	117.4 (CH)	4.63, *ddd* (8.5, 1.5, 1.5)	122.4 (CH)	5.13, *ddd* (7.5, 1.5, 1.5)
14'	137.8 (qC)		133.6 (qC)	
Me-15	18.6 (CH_3_)	1.83, 3H, *d* (1.5)	17.8 (CH_3_)	1.69, 3H, *d* (1.5)
Me-16	26.0 (CH_3_)	1.54, 3H, *d* (1.5)	25.7 (CH_3_)	1.60, 3H, *d* (1.5)
Me-15'	18.7 (CH_3_)	1.61, 3H, *d* (1.5)		
Me-16'	25.8 (CH_3_)	1.72, 3H, *d* (1.5)		
OH-5		12.29, *s*		12.46, *s*
OH-8		12.48, *s*		12.56, *s*
OH-5'		12.40, *s*		
OH-8'		12.07, *s*		

*^a^* Spectra recorded at 500 MHz in CDCl_3_ for ^1^H and 125 MHz for ^13^C; *^b^* The attached protons were determined by DEPT and HMQC experiments; *^c^* The *J* values are in Hz in parentheses. The chemical shift values (δ_H_/δ_C_) are in ppm downfield from TMS.

From the above findings, compound **2** was identified as a novel heptacyclic arnebin-7 dimer. Comparison of proton chemical shifts and *J* values of H-12, H-11' and H-12' of **2** with those of **5** (Table 2), suggested the relative stereochemistries at C-12, C-11' and C-12' to be the same. The *^4^J* long-range (*J =* 2.5 Hz) coupling occurred between H-2 and H-12' disclosed the *cis* configuration of H-2 [17]. Compound **2** was thus proposed to be 2R*, 3S*,11R*,11'R*, 12S*,12'S*-configured and named arnebidin (Figure 1).

**Figure 5:**
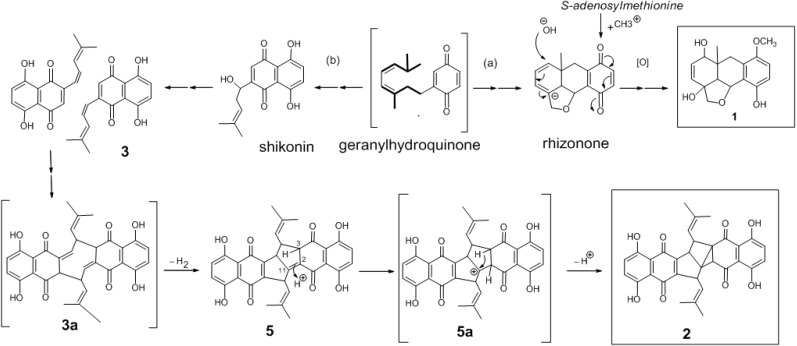
Proposed biosynthetic pathways for **1** and **2**.

The new metabolite **1** is proposed to be biosynthetically derived from a monoterpenoid benzoquinone (rhizonone) [18], obtained by cyclization of an intermediate precursor (geranylhydroquinone), through hydroxylation of C-5, oxidation of C-8, and monomethylation of the *p*-quinonoid moiety (pathway a). The production of monomeric metabolites from geranylhydroquinone e.g., shikonin and arnebin-7 (**3**) (pathway b), followed by condensation of two molecules of **3** from their side chain was suggested to yield the intermediate **3a** then the hexacyclic dimeric metabolite shikometabolin D (**5**) with a bicylic pentalene core. Protonation of metabolite **5** at C-2 affords the carbonium ion **5a**. The subsequent deprotonation at C-3 of **5a** and the following electrophilic aliphatic substitution at C-11could generate the heptacyclic derivative (**2**) with a tricyclo[3.3.0.0^1,3^]octane core. The presence of 2,11-double bond is thought to catalyze the intramolecular rearrangement (cyclopropanation) in metabolite **5** to yield compound **2**. Although they are few in number, cyclopropyl-containing natural products, in which the double bonds of their precursors involved in the cyclopropanation by a similar mechanism, have been reported. These can be exemplified by the biosynthesis of crythanthemic acid from dimethylallyl diphosphate [19] and sabinene or thujone from terpinen-4-yl cation [20] and substantiated the proposed biosynthetic pathway for arnebidin (**2**) (Figure 5).

## 3. Experimental

### 3.1. General

IR and UV spectra were recorded on Hitachi I-2001 infrared and Hitachi U-3210 spectrophotometers, respectively. Mass spectral data were obtained by EI and FAB with a VG Quattro GC/MS spectrometer. HRMS spectra were obtained by ESI on a Bruker APEX II mass spectrometer. NMR spectra were recorded on Brukers Avance DPX-300 and 400 and Varian INOVA-500 NMR spectrometers at 300, 400, and 500 MHz for ^1^H, and at 75, 100, and 125 MHz for ^13^C, respectively, in CDCl_3_ or CD_3_OD. Silica gel 60 (Merck, 230–400 mesh) were used for column chromatography. Precoated Si gel plates (Merck, Kieselgel 60 F_254_, 0.25 mm) were used for analytical thin layer chromatography (TLC). Preparative high-performance liquid chromatography (HPLC) was performed on a Hitachi L-7100 apparatus using a Merck Hibar Si-60 column (250 × 21 mm, 7 μm) and Hitachi L-7400 UV detector (detection wavelength = 280 nm).

### 3.2. Plant Materials

The roots of *A. hispidissima* were collected from Wady Khashaba, South of Sinai Peninsula, Egypt, during spring, and identified by Ibrahim A. Mashaly, Department of Botany, Faculty of Science, MU. A voucher sample (S-98-1) was deposited at the Department of Pharmacognosy, Faculty of Pharmacy, MU. All freshly prepared organic extracts and fractions of the air-dried roots were kept in freezer at −20 °C until use.

### 3.3. Extraction and Isolation of Compounds

The air-dried roots of *A. hispidissima* (1 Kg) were powdered and exhaustively extracted with light petroleum (boiling point 60–80 °C). The light petroleum extract was concentrated under vacuum to afford a reddish brown viscous residue (13.8 g). The marc was exhaustively extracted with MeOH and the solvent-free extract (106.7 g) was then successively partitioned with H_2_O/*n*-hexane, H_2_O/CH_2_Cl_2_ and then with H_2_O/EtOAc. The three organic partitions were separately evaporated under vacuum to give the MeOH-derived *n*-hexane, CH_2_Cl_2_, and EtOAc fractions (MHF: 4.9 g, MCF: 3.6 g, and MEF: 6.5 g, respectively). The light petrol extract was chromatographed on a column of Si gel and eluted with benzene in *n*-hexane then CH_2_Cl_2_ in benzene (0%–100%, gradient) to afford six fractions (P1–P6). The orange red fractions P2 eluted with benzene-*n*-hexane (1:1 to 7:3, gradient) and P5 eluted with benzene-*n*-hexane (1:0) were purified separately on preparative Si gel TLC using benzene-*n*-hexane (2:3, double run) or CH_2_Cl_2_-benzene-acetone (50:50:1) to obtain **3** (6 mg) from P2 and **2** (7 mg) from P5. MCF was chromatographed on Si gel column using MeOH in CH_2_Cl_2_ (0% to 2%) to give a UV-absorbing fraction. This fraction, eluted with 2% MeOH in CH_2_Cl_2_, was then purified on Si gel column using 1.5% MeOH in CH_2_Cl_2_ followed by preparative Si gel HPLC using 1.5% MeOH in CH_2_Cl_2_ (flow rate 2.5 mL/min) to yield **1** (3.2 mg). MEF was fractionated on Si gel column using EtOAc-MeOH-H_2_O (95:5:0.5 to 60:40:4, gradient) to obtain a UV-absorbing fraction which was further purified by preparative Si gel TLC using 12% MeOH in CH_2_Cl_2_ to afford **4** (2.5 mg).

*Compound* **1***.* Colorless feathery crystals, sublimable at 190–191 °C. TLC (Si gel, solvent: MeOH-CH2Cl2 [1:19], *R_f_* 0.17). UV λ_max_ (CHCl_3_) 281 nm. IR (neat) ν_max_ 3356 (broad), 2960, 2838, 1601, 1510, 1458, 1408, 1272, 1093, 1030 cm^−1^. ^1^H and ^13^C-NMR data (CDCl_3_), see Table 1. FAB-MS *m*/*z*: 327 (12.3, [M+Na]^+^), 305 (26.8, [M+H]^+^), 304 (100, [M]^ +^), 287 (17.3, [M-H_2_O+H]^+^), 269 (8.9, [M-2 H_2_O+H]^+^), 239 (11.9 [M-H_2_O-MeOH-Me]^+^), 241 (12.5 [M-H_2_O-CH_2_O-Me]^+^), 189 (26.0, [M-3H_2_O-MeOH-CH_2_O+H]^+^). EI-MS 70 eV *m*/*z*: 304 (38.8, [M]^+^), 286 (3.4, [M-H_2_O]^+^), 268 (11.9, [M-2H_2_O]^+^), 239 (12.4, [M-H_2_O-MeOH-Me]^+^), 188 (50.0, [M-3H_2_O-MeOH-CH_2_O+H]^+^). HRFAB-MS *m*/*z* 304.1307 (calcd for C_17_H_20_O_5_, 304.1311).

*Acetylation of compound* **1**: A solution of 1 (2.2 mg, 0.007 mM) in pyridine (0.3 mL) was mixed with Ac_2_O (0.2 mL), and the mixture was stirred at RT for 24 h. The mixture was diluted to 5 mL with distilled water, neutralized with sodium carbonate powder, extracted with ether (2 × 3 mL). The ether extract was then evaporated to dryness, dissolved in acetone*-n*-hexane (1:3) and subjected to normal phase HPLC using the same solvent system at a flow rate of 1 mL/min. Two UV-absorbing products: a diacetyl derivative **1a** (1.2 mg, 0.0031 mmol, 42.7%) and a triacetyl derivative **1b** (0.6 mg, 0.0014 mmol, 19.3%) were separately collected. 

**1a**: A white amorphous powder. TLC (Si gel, solvent: acetone-*n*-hexane [1:3], *R_f_* 0.11). IR (neat) ν_max_ 3350, 2950, 2830, 1770, 1734, 1481, 1369, 1240, 1201, 1084 cm^−^^1^. ^1^H-NMR (300 MHz, CDCl_3_) δ_H_: 6.95 (1H, d, *J* = 8.8 Hz, H-2), 6.84 (1H, d, *J* = 8.8 Hz, H-3), 6.07 (2H, m, H-6 and H-7), 5.23 (1H, d, *J* = 5.1 Hz, H-9), 4.93 (1H, br s, H-5), 3.99 (1H, d, *J* = 10.0 Hz, Hα-12), 3.92 (1H, d, *J* = 10.0 Hz, Hβ-12), 3.82 (3H, s, MeO-4), 2.73 (1H, d, *J* = 15.6 Hz, Hβ-10), 2.49 (1H, d, *J* = 15.6 Hz, Hα-10), 2.47 (1H, d, *J* = 5.1 Hz, H-8a), 2.33 (3H, s, AcO-1), 2.11 (3H, s, AcO-5), 0.98 (3H, s, Me-11), ^13^C-NMR (75 MHz, CDCl_3_) δ_C_: 170.5 (1C, qC, 5-OAc), 170.4 (1C, qC, 1-OAc), 155.0 (1C, qC, C-4), 144.0 (1C, qC, C-1), 133.0 (1C, CH, C-7), 127.1 (1C, qC, C-9a), 126.0 (1C, CH, C-6), 125.8 (1C, qC, C-4a), 120.5 (1C, CH, C-2), 110.1 (1C, CH, C-3), 78.8 (1C, qC, C-8), 77.9 (1C, CH_2_, C-12), 72.7 (1C, CH, C-9), 71.4 (1C, CH, C-5), 55.6 (1C, CH_3_, 4-OMe), 50.5 (1C, CH, C-8a), 34.0 (1C, qC, C-10a), 33.0 (1C, CH_2_, C-10), 21.1 (2C, CH_3_, 1-OAc and 5-OAc), 20.0 (1C, CH_3_, C-11). EI-MS 70 eV *m*/*z*: 388 (1.7, [M]^+^), 370 (2.3, [M-H_2_O]^+^), 328 (1.7, [M-AcOH]^+^), 295 (32.2 [M-AcOH-H_2_O-Me]^+^), 268 (7.7, [M-2AcOH]^+^), 253 (49.4, [M-2AcOH-Me]^+^), 239 (12.5).

**1b**: A white amorphous powder. TLC (Si gel, solvent: acetone-*n*-hexane [1:3], *R_f_* 0.22). IR (neat) ν_max_ 2950, 2830, 1770, 1734 (strong), 1479, 1371, 1240, 1201, 1024 cm^−1^. ^1^H-NMR (300 MHz, CDCl_3_) δ_H_: 6.95 (1H, d, *J* = 8.8 Hz, H-2), 6.85 (1H, d, *J* = 8.8 Hz, H-3), 6.61 (1H, d, *J* = 10.0 Hz, H-7), 6.10 (1H, dd, *J* = 10.0, 5.6 Hz, H-6), 5.05 (1H, d, *J* = 5.0 Hz, H-9), 4.90 (1H, d, *J* = 5.6 Hz, H-5), 4.40 (1H, d, *J* = 11.5 Hz, Hα-12), 4.05 (1H, d, *J* = 11.5 Hz, Hβ-12), 3.83 (3H, s, MeO-4), 2.73 (1H, d, *J* = 16.4 Hz, Hβ-10), 2.54 (1H, d, *J* = 16.4 Hz, Hα-10), 2.83 (1H, d, *J* = 5.0 Hz, H-8a), 2.30 (3H, s, AcO-1), 2.12 (3H, s, AcO-8), 2.10 (3H, s, AcO-5), 0.97 (3H, s, Me-11), ^13^C-NMR (75 MHz, CDCl_3_)δ_C_: 170.7 (2C, qC, 5-OAc and 8-OAc), 170.5 (1C, qC, 1-OAc), 155.0 (1C, qC, C-4), 144.0 (1C, qC, C-1), 130.0 (1C, CH, C-7), 129.0 (1C, CH, C-6), 128.0 (1C, qC, C-4a), 127.1 (1C, qC, C-9a), 120.5 (1C, CH, C-2), 110.2 (1C, CH, C-3), 87.1 (1C, qC, C-8), 76.0 (1C, CH_2_, C-12), 72.6 (1C, CH, C-9), 71.1 (1C, CH, C-5), 56.3 (1C, CH_3_, 4-OMe), 47.1 (1C, CH, C-8a), 34.0 (1C, qC, C-10a), 33.0 (1C, CH_2_, C-10), 22.2 (1C, CH_3_, 8-OAc), 21.1 (2C, CH_3_, 1-OAc and 5-OAc), 18.3 (1C, CH_3_, C-11). EI-MS 70 eV *m*/*z*: 370 (2.6, [M-AcOH]^+^), 310 (1.1, [M-2AcOH]^+^), 295 (32.2 [M-2AcOH-Me]^+^), 253 (28.4), 250 (3.9, [M-3AcOH]^+^), 241 (17.6), 227 (26.7). 

*Compound* **2***.* Orange red amorphous powder. TLC (Si gel, solvent: benzene-CH_2_Cl_2_-acetone [50:50:1], *R_f_* 0.34). ^1^H and ^13^C-NMR data (CDCl_3_), see Table 2. EI-MS 70 eV *m*/*z*: 538 (100, [M]^+^), 523 (7.9, [M-CH_3_]^+^), 495 (7.8, [M-isopropyl]^+^), 469 (12.2, [M-isopentenyl]^+^), 457 (8.5, [M-isohexenyl]^+^), 415 (6.0), 403 (10.6, [M-C_7_H_4_O_3_-H]^+^), 391 (4.0), 202 (4.0, [C_11_H_6_O_4_]^+^), 391 (5.0), 317 (3.0), 279 (3.5), 239 (3.0), 202 (4.0, [C_11_H_6_O_4_]^+^), 189 (4.0, [C_10_H_5_O_4_]^+^), 149 (6.5), 108 (4.0, [C_6_H_4_O_2_]^ +^).

*Compound* **3**: Reddish violet amorphous powder, m.p. 94–95 °C. TLC (Si gel, solvent: benzene-CH_2_Cl_2_-acetone [50:50:1], *R_f_* 0.82). ^1^H and ^13^C-NMR data (CDCl_3_), see Table 2. EI-MS 70 eV *m*/*z*: 272 (100, [M]^+^), 254 (15.5, [M-H_2_O]^+^), 229 (37.2), 216 (37.1, [M-2CO]^+^), 204 (98.4, [M-C_5_H_8_]^+^), 189 (3.7, [M-C_10_H_5_O_4_]^+^), 108 (4.0, [M-C_6_H_4_O_2_]^+^).

*Compound* **4**: White amorphous powder; m.p. 210–212 °C. TLC (Si gel, solvent: MeOH-CH2Cl2 [6:94], *R_f_* 0.36). ^1^H-NMR (400 MHz, CD_3_OD) δ_H_: 7.60 (1H, d, *J* = 2.0 Hz, H-2), 7.51 (1H, dd, *J* = 8.0, 2.0 Hz, H-6), 6.90 (1H, d, *J* = 8.0 Hz, H-2), 3.87 (3H, s, OCH_3_). ^13^C -NMR (100 MHz, CDCl_3_) δ_C_: 174.0 (1C, qC, C-7), 150.4 (1C, qC, C-4), 148.2 (1C, qC, C-3), 129.6 (1C, qC, C-1), 124.4 (1C, CH, C-6), 115.3 (1C, CH, C-5), 114.1 (1C, CH, C-2), 56.7 (1C, CH_3_, OCH_3_). EI-MS 70 eV *m*/*z* (intensity): 530 (2.9, [M]^+^), 194 (35.0, [C_10_H_10_O_4_]^+^), 177 (36.5), 150 (15.4). FAB-MS *m*/*z*: 553 (0.7, [M+Na]^+^), 531 (1.0, [M+H]^+^), 530 (1.0, [M]^+^), 195 (9.4, [C_11_H_6_O_4_+H]^+^), 194 (10.7, [C_11_H_6_O_4_]^+^), 177 (57.1).

## 4. Conclusions

The phytochemical study of *Arnebia hispidissima* (Lehm.) DC. roots resulted in the isolation two new polycyclic gernaylhydroquinone-derived metabolites: arnebacene (**1**) and arnebidin (**2**). Compound **1** was defined as (5S*,8R*,8aS*,9S*,10aR*)-1,5,8-trihydroxy-4-methoxy-10a-methyl-8,9-oxymethylene-5,10a,8,8a,9,10-hexahydroanthracene. Compound **2** is reported herein as an heptacyclic arnebin-7 dimer with an unprecedented tricyclo[3.3.0.0^1,3^]octane core formed by the coupling of side chains.
